# Parent attitudes towards predictive testing for autism in the first year of life

**DOI:** 10.1186/s11689-024-09561-w

**Published:** 2024-08-17

**Authors:** Aurora M. Washington, Amanda H. Mercer, Catherine A. Burrows, Stephen R. Dager, Jed T. Elison, Annette M. Estes, Rebecca Grzadzinski, Chimei Lee, Joseph Piven, John R. Pruett, Mark D. Shen, Benjamin Wilfond, Jason Wolff, Lonnie Zwaigenbaum, Katherine E. MacDuffie

**Affiliations:** 1 University of North Carolina at Chapel Hill, Chapel Hill, NC USA; 2 Portland State University, Portland, OR USA; 3 University of Minnesota, Minneapolis, MN USA; 4 University of Washington, Seattle, WA USA; 5 Washington University School of Medicine in St. Louis, St. Louis, MO USA; 6 Seattle Children’s Research Institute, M/S JMB-6, PO Box 5371, Seattle, WA 98145 USA; 7 University of Alberta, Edmonton, Alberta Canada

**Keywords:** Autism, Biomarkers, Prediction, Stakeholder engagement, Bioethics

## Abstract

**Background:**

Emerging biomarker technologies (e.g., MRI, EEG, digital phenotyping, eye-tracking) have potential to move the identification of autism into the first year of life. We investigated the perspectives of parents about the anticipated utility and impact of predicting later autism diagnosis from a biomarker-based test in infancy.

**Methods:**

Parents of infants were interviewed to ascertain receptiveness and perspectives on early (6-12 months) prediction of autism using emerging biomarker technologies. One group had experience parenting an older autistic child (*n*=30), and the other had no prior autism parenting experience (*n*=25). Parent responses were analyzed using inductive qualitative coding methods.

**Results:**

Almost all parents in both groups were interested in predictive testing for autism, with some stating they would seek testing only if concerned about their infant’s development. The primary anticipated advantage of testing was to enable access to earlier intervention. Parents also described the anticipated emotions they would feel in response to test results, actions they might take upon learning their infant was likely to develop autism, attitudes towards predicting a child’s future support needs, and the potential impacts of inaccurate prediction.

**Conclusion:**

In qualitative interviews, parents of infants with and without prior autism experience shared their anticipated motivations and concerns about predictive testing for autism in the first year of life. The primary reported motivators for testing—to have more time to prepare and intervene early—could be constrained by familial resources and service availability. Implications for ethical communication of results, equitable early intervention, and future research are discussed.

**Supplementary Information:**

The online version contains supplementary material available at 10.1186/s11689-024-09561-w.

Recent years have seen considerable research investment in technologies that aim to predict a later diagnosis of autism spectrum disorder (hereafter autism) from information obtained in the first year of life. Technologies such as MRI, EEG, eye-tracking, and digital phenotyping are being investigated for autism prediction prior to 12 months, with initial work in infant and toddler-age samples showing high predictive accuracy (see Table 1) [1, 2]. These emerging technologies create possibilities for developing and testing very early interventions that capitalize on the period of rapid brain growth, brain plasticity, and skill acquisition in the first year of life [3]. However, the evidence base for very early intervention is nascent, with three recent systematic reviews/meta-analyses reporting small, non-significant impacts on clinically relevant features [4–6]. Emerging biomarker technologies, therefore, raise critical ethical questions about the potential benefits and harms of autism prediction in infancy [7, 8], alongside practical questions about how to best support families who learn their infant is highly likely to develop autism months to years before symptoms consolidate into a diagnosable behavioral syndrome [3, 9].

**Table 1:** Emerging predictive technologies. Table includes examples of completed and ongoing prospective studies using biomarker technologies to capture biological or behavioral differences in the first years of life that predict subsequent autism diagnosis. PPV = positive predictive value; NPV = negative predictive value

Lead Author/Contact PI	Year published/awarded	Journal/Grant#	Method	Population	Age	Sensitivity	Specificity	PPV	NPV
**Prediction in first year of life (0-12 mo)**									
Wolff	2015	Brain	Diffusion tensor imaging	Infant siblings (n = 270) and controls (n = 108)	6 mo			65-68%	85-85%
Hazlett	2017	Nature	Structural MRI	Infant siblings (n = 106) and controls (n=42)	6-12 mo	88%	95%	81%	97%
Emerson	2017	Science Translational Medicine	Functional MRI	Infant siblings (n = 59)	6 mo	81.80%	100%	100%	96%
Shen	2013; 2017	Brain; Biological Psychiatry	MRI (extra-axial cerebrospinal fluid)	Infant siblings (n = 33; n = 221) and controls (n = 22; n = 122)	6 mo	66-80%	67-68%	36-62%	83-88%
Bosl	2018	Scientific Reports	EEG entropy	Infant siblings (n = 99) and controls (n = 89)	3 mo	82%	99%	97%	-
Gabard-Durnam	2019	Nature Communications	Frontal EEG spectral power	Infant siblings (n = 102) and controls (n = 69)	3-12 mo	82%	86%	72%	92%
**Prediction in older infants/toddlers ( > 12 mo)**									
Perochon	2023	Nature Medicine	Digital behavioral phenotyping	General population (pediatric primary care; n = 475)	17-36 mo	87.80%	80.80%	97.80%	40.60%
Jones	2023	JAMA	Eye tracking	General population (autism specialty clinics; n = 475)	16-30	71%	80.70%	76.20%	76.20%
Jones	2023	JAMA Network Open	Eye tracking in two cohorts (discovery and replication)	General population (autism specialty clinics; n = 1089 total, 719 in discovery, 370 in replication)	16-45	80.6-	82.3-	81.9-87.3%	81-85.4%
						81.9%	89.9%		
**Active NIH grants replicating/extending prior work for prediction in infancy**									
Wetherby	2018	R01MH121364	Mobile screening app	General population (pediatric primary care)	2-24 mo				
Pruett	2019	R01MH118362	Structural and functional MRI	Infant siblings	6 mo				
Dawson	2019	R01MH121329	Digital phenotyping via app	General population	6-12 mo				
Jones	2019	R01MH121363	Eye tracking	General population (pediatric primary care)	9 mo				
Ozonoff	2019	R01MH121344	Video coding	General population	0-12 mo				
Jeste	2021	R01MH121462	EEG and eye tracking	Infant siblings	6, 12 mo				
Nelson	2021	R01NS120986	Resting EEG	General population (pediatric primary care)	4, 9, 12 mo				
Sheinkopf	2022	R01MH121345	Infant cry acoustics	General population	0-12 mo				

Key insights into the potential benefits and harms of autism prediction in infancy can be gleaned from directly engaging parents, who will be tasked with deciding whether to seek predictive biomarker testing for their children once such tools are available. In a prior qualitative interview study (*n* = 14) of parent attitudes towards early detection of autism, parents of infants at high likelihood for autism described the potential benefits of an earlier diagnosis for adjusting their parenting approach and expectations for their child, and the potential risks of encountering stigma associated with an autism diagnosis [8]. However, this prior study, which was conducted in Belgium, did not address the potential impacts of a predictive diagnosis for navigating autism services—an issue of particular salience in the context of the US healthcare system which is decentralized and characterized by long delays and disparities in the receipt of autism-specific services following a standard diagnosis [10–12].

We sought to build upon this prior work to understand the attitudes of two groups of parents of infants aged 6-13 months: those with and without prior experience parenting a child with autism. The autism experience (AE) parents were recruited from a longitudinal neuroimaging study of infant siblings of autistic children (approximately 20% of infant siblings go on to develop autism, and this population has been the focus of most published predictive biomarker work to date) [13]. This group of parents, already enrolled in research, is not representative of all parents of infants and an older child or children with autism. Instead, they represent the type of parents who may be “early adopters” of new technologies currently under development to predict autism in infancy.

Given the goal to extend predictive testing beyond the infant sibling context (1,2; Table 1), we were also interested in the perspectives of parents with no autism experience (NAE), who have infants at low likelihood of developing autism (i.e., no family history of autism). We recruited NAE parents from a general pediatrics practice in Seattle, USA. This group lacked geographic diversity and was not intended to be representative of all US parents. Rather, we sought to gain an in-depth understanding of the perspectives of a group of parents who, by virtue of being in the same pediatrics practice, experienced the same routine developmental screening procedure for their infants. This provided a naturally occurring context in which the NAE families shared a similar experience with autism screening for their same-aged infants but could vary regarding their perspectives on the potential of new technologies to enable a predictive diagnosis of autism in infancy. By employing a similar set of interview questions with both AE and NAE parents, we sought to understand the perspectives of parents with relevant experience and those naïve to the autism diagnosis/services landscape with the goal to inform and guide future clinical translation of biomarker-based prediction in infancy.

## Methods

Fifty-five semi-structured phone interviews were conducted with two groups of parents (30 interviews with AE parents and 25 interviews with NAE parents; see participant demographics in Table 2). Eligibility required having an infant age 6-13 months at the time of the interview and the ability to participate in the interview in English. We used a purposeful sampling approach, with the goal of understanding a specific phenomenon (how parents of infants at high and low likelihood for autism think about predictive testing), but not aiming to generalize from this small studied sample to the general population[14]. Sample size was determined a-priori (we originally planned for 25-30 interviews in each group) to ensure adequate group size to qualitatively compare responses across groups [15].

**Table 2:** Demographics characteristics of parents

	Total (*n*=55)	Parents with autism experience (AE; *n*=30)	Parents with no autism experience (NAE; *n*=25)
Gender			
Female	52	29	23
Male	4	3	1
Non-Binary/ Queer	1	0	1
Race			
White	48	29	19
Black	3	2	1
Asian	4	0	4
Native American	1	0	1
Two or More	1	1	0
Ethnicity			
Hispanic	12	9	3
Non-Hispanic	45	23	22

In two AE interviews, two parents participated. We have thus reported demographics for 32 parents who participated in 30 AE interviews. All NAE interviews were with individual parents

### AE parent recruitment

Study procedures for all Infant Brain Imaging Study – Early Prediction (IBIS-EP; R01MH118362) sites were approved by the Washington University in St. Louis sIRB for multi-site research. Eligible parents were recruited for qualitative interviews following their 6-month study visit, which included behavioral assessment and MRI scanning, but prior to receiving any feedback from the visit. Thirty AE interviews (with 32 parents; two interviews included both the child’s mother and father) were completed between January 2020 and January 2022. Due to the distributed nature of recruitment across multiple IBIS-EP study sites, we are not able to calculate response rate for this group. Interviews ranged from 24-79 minutes, with an average duration of 45 minutes.

### NAE parent recruitment

Study procedures were approved by the Seattle Children’s IRB. Invitations to participate were mailed to caregivers of infants seen at a single pediatrics practice within the eligible age range (6-12 months; some infants were 13 months old by the time the interviews occurred). From 77 invitations, 31 parents (40%) agreed to participate. Four were lost to contact, 1 stopped the interview early, and 1 interview was not recorded due to technical difficulties. Twentyfive NAE interviews, completed between June and October 2021, were included in analyses. Interviews ranged from 30-72 minutes, with an average duration of 43 minutes. Although this group had no prior autism parenting experience, 20/25 had at least one older child in addition to their infant, parallel to the AE parent group who also had at least one older child.

### Semi-structured interview procedure

Separate semi-structured interview guides (available in the Supplement) were used to assess AE and NAE parent attitudes towards predictive testing (with MRI offered as the primary example) for autism between 6-12 months of age. Interview guides were developed based upon prior qualitative work with parents of infants at high likelihood for developing autism [16], with input from our interdisciplinary co-author team.

At the start of the interview, the interviewer briefly described a potential predictive MRI test that “could tell between 6-12 months whether or not your infant is likely to develop autism”. Specifics of test accuracy (e.g., sensitivity, specificity) were not provided due to evidence that patients have a difficult time realistically understanding such probabilities [17]. Interviews were conducted by phone by a licensed clinical psychologist and experienced qualitative interviewer (KM). For AE parents, the interview was framed as “related to but separate from” their participation in IBIS-EP; however, it is possible that the interviewer’s affiliation with the study influenced parent responses in this group. Phone interviews were audio recorded and transcribed for analysis.

### Analysis

We used an atheoretical analytic approach—qualitative content analysis—which is common in empirical bioethics and health sciences research [18]. This is an appropriate analysis method when the informational content of the interviews is of primary interest. As an interdisciplinary co-author team, with training in bioethics, biotechnology, clinical and educational psychology, child psychiatry, radiology, and neuroscience, we approached these interviews in an exploratory manner, without the intention to generate or test theory, but rather to understand the complex, contextual, constructed, and subjective reality experienced by interviewees [19], and how this reality could be influenced by earlier knowledge that their child is (or is not) likely to develop autism. It was our hope that the interviews would provide insights into considerations for clinical translation of predictive biomarker tools for autism that might not have otherwise occurred to the scientists working to develop such tools.

Transcripts were entered into Atlas.ti. A starting code list made up on deductive codes (derived from interview guide questions) was supplemented by “in-vivo” codes generated from additional concepts raised in the interviews. The AE parent transcripts were coded first. The interviewer and secondary coders independently coded the first set of three transcripts; the team then met to discuss and revise the codebook. Two coders applied the revised codebook to 70% of transcripts, discussing to resolve discrepancies, and the remaining 30% of transcripts were single-coded. The NAE parent transcripts were coded second, using the AE codebook as a starting point, and we repeated the process of the interviewer and secondary coders reviewing three NAE transcripts to start and adding new in-vivo codes to capture aspects of the interview that differed across the two groups. The remaining NAE transcripts were coded using the same procedure as the AE transcripts.

The data presented here are a subset of all the content covered in the interviews. We included only the portion of the data that answered the following research questions, which we felt were of the highest priority to inform clinical translation:

1. Are parents interested in testing that would predict a categorical future autism diagnosis?
2. What do parents anticipate to be the impacts of learning that their infant is likely to develop autism?
3. Are parents interested in testing that would predict a child’s future level of functioning or support needs?
4. What do parents anticipate to be the impacts of an inaccurate prediction?

To answer these questions, we revisited transcript segments that were coded with relevant codes and derived both quantitative and qualitative summaries. For three of our research questions, we calculated a quantitative summary of response frequencies for each group to ease direct comparison (Table 3) by categorizing coded responses from the interviews. Qualitative summaries were derived via close reading of coded segments and, in some cases, secondary coding and reorganization, followed by descriptive and interpretive summaries written as memos [18]. We present results related to each of our four research questions below, with related qualitative summaries and supporting quotes provided for each.

**Table 3:** Quantitative summary of qualitative results

	Parents with autism experience (AE; *n*=30)		Parents with no autism experience (NAE; *n*=25)	
Interest in categorical/diagnostic prediction				
Yes	20	97%	17	68%
Yes, if concerned	1	3%	7	28%
No	0	0%	1	4%
Interest in prediction of a child’s future support needs				
Yes	17	59%	20	83%
No	6	21%	1	4%
Ambivalent	6	21%	3	13%
Not asked	1	--	1	--
Judgement of which type of inaccurate result would cause most stress				
False negative	19	79%	16	70%
False positive	3	13%	6	26%
Ambivalent	2	8%	1	4%
Not asked	6	--	2	--

Percentages do not include parents who were not asked a given question. Percentages may not total 100 due to rounding

## Results

### Interest in predictive testing for autism

Parents were asked if they would be interested in undergoing predictive testing to learn whether their infant would develop autism. Almost all AE parents and most NAE parents said they would pursue testing if available, with a smaller number saying they would want testing only if concerned about their child’s development (Table 3). As one parent noted, “If there was strong, maybe family history of it, I believe that I would. But since I don’t have that, I don’t think I would want to know (NAE-7).” Interested parents described the benefits of the additional time offered by a predictive test to mentally prepare and gather information. As one noted: “If I have time to prepare, it would be better than if it came as a shock, you know, I could read up about it…just have the information so that, you know, when the autism does start to be more visible, or affect her life more, then I’d be prepared to handle that (NAE-25).” Others mentioned it would be nice to know earlier to process emotions: “I guess just as a parent, I’d have more time to kind of like cope and have that grief period…grieving the neurotypical experience and accepting the neurodivergent experience (NAE-19).” Some parents mentioned the utility of having more time to enroll in services for their child, saying things like: “Everything is a waiting game when it comes to getting into different therapies, to get the official diagnosis…And so I think the sooner that you can find out that your child has it, the sooner you can start getting them help (AE-25).”

None of the AE parents and only one NAE parent said under no circumstances would they pursue predictive testing. This parent saw no value in identifying autism prior to symptoms, noting: “I wouldn’t ever want to feel like I’m kind of like jumping the gun and addressing needs that my child doesn’t have yet. I’d rather just address the child in front of me versus the child that could be in front of me (NAE-23).”

### Anticipated impact of predictive testing

Parents were asked to consider what the impact of a predictive test might be on their families. In response to a positive autism prediction, most parents said that they would try to get their child into therapy or onto waitlists as early as possible. AE parents anticipated they would draw on their prior experience parenting their older autistic child. As one said, “I’d have a plan of action…And I wouldn’t have a lot of expectations of [infant] like milestones and stuff, I would just let him be like [older child] and just kind of go with the flow (AE-23).” Some parents questioned at what age children could start therapy: “I mean, obviously, you can’t start ABA [applied behavior analysis] at six months, but whatever it is that you can, speech, or whatever it is that you can do ahead of time, I’d want that in place (AE-26).” A few parents anticipated making lifestyle changes, such as relocating from a rural area to a larger city with more resources to support their child. NAE parents anticipated seeking information from their pediatrician about next steps and looking for support groups and information online.

While most parents were interested in predictive testing, many expressed concerns about identifying autism at such an early age. One parent noted: “I would feel like, okay, [child]’s, labeled as autistic. And whatever support that brings her is like the most important thing, but it could also mean she’s discriminated against. And it would be harder to remove that label (AE-8).” Another parent expressed their concerns about the emotional impact on parents of a diagnosis, saying, “Maybe if someone’s in a really, really dark place already in their life, depression or in crisis mode based on whatever is happening… it might not be good to the mental health of certain people. Based on timing (AE-22).” In addition, some parents felt that a predictive autism diagnosis could limit the expectations that they or others hold for their child. As one parent said, “If there’s something in her chart that says, you know, ‘diagnosed with autism by MRI’, then I just feel like…her care would be different…and then I wouldn’t want myself and like my family to treat her any differently knowing that, you know, it’s kind of like a self-fulfilling prophecy. So then I would always wonder…are we enabling the autism by accommodating more for it?…what would it be like if we didn’t diagnose it so early on (NAE-18)?”

### Attitudes towards prediction of support needs, not just diagnosis

Parents were told that a goal of current research is to develop technologies to predict not just categorical autism diagnosis, but a child’s anticipated level of support needs. Most parents in both groups (Table 3) said that predicting a child’s level of support needs would be beneficial. As one parent shared, “we can better plan our life. I’m kind of a planner…for example, if they were going to maybe have some more like physical handicaps then I think we would need a different house (AE-18).” Another parent shared, “I think it’s super important to know, because the biggest thing is getting the kids the resources that they need…if your child does have larger needs…then it just pulls that band-aid off quicker, you know, so that you’re prepared for that (AE-7).” Some parents spontaneously mentioned the utility of knowing in which specific domains (i.e., language, cognitive, motor, or sensory functioning) their child was likely to need the most support.

A smaller group of parents did not see utility in learning about future support needs. One said, “as a worrier, it’s almost like the more information that I know, without seeing it manifested in front of me, the worse my wheels spin... So, I think just a simple [diagnostic] yes or no, I would be more interested in (NAE-11).” Other parents were concerned about more specific expectations limiting their child’s potential: “I think, if somebody told me that he was going to be severely autistic, I would lose all hope and not put as much effort in (AE-1).” As another parent put it: “Don’t just give up on his speech because you think that that, oh, he's gonna be low functioning, you know, don’t give up on…trying to help him in certain areas, because you think you think that he might not make progress. Don’t limit him (AE-6).”

### Anticipated impact of receiving an inaccurate prediction

Parents were asked to imagine what it would be like and how they would handle two types of inaccurate results: 1) A false positive (being told your child will develop autism but they never do) or 2) a false negative (being told your child will develop typically but they end up with an autism diagnosis).

Anticipating a false positive result was associated with emotions like frustration and annoyance for most parents. As one said, “that would be such an emotional roller coaster, and I’d probably be a little bit frustrated (NAE-4).” Some parents were concerned with time and resources being wasted: “I would have gone through that struggle…like putting them in programs and waitlists and having them evaluated and dealing with insurance here and there, and all of that. So, I’d be a little annoyed that I went to that, or through that struggle for no reason (AE-22).” Other parents noted that delivery of unnecessary services would not harm their child: “Part of me thinks that I would be mad, but in all actuality, I don’t know if I would, because what’s the problem with extra therapies and focusing on speech (AE-7)?”

Anticipating a false negative result was associated with stronger negative emotions for most parents. As one parent noted: “[it] would be super frustrating because … you’ve opted to go with the (let’s be honest) probably a little bit more of an expensive test that may not be covered by insurance...and then you go and do that and find out that it’s actually inaccurate (NAE-15).” Parents additionally anticipated feeling that they had missed a time-limited opportunity to be providing their child with services: “It would cause me anxiety in the sense that, like, did we miss this crucial window of like, early development, that would have made a difference or could have made a difference? And then I think we would just race to play catch up, like as much as possible (AE-30).”

Due to concerns about lost time for intervention, most parents (Table 3) felt that a false negative would be harder than a false positive: “I would be more upset…especially if we missed the window of being able to do something about it (NAE-17).‬” ‬‬A smaller portion of parents felt that a false positive would be more stressful, given the emotional toll of waiting for autism characteristics to emerge: “if I was told that she was going to get it, and…with each passing day, she’s not showing any signs, I would just continue to wait for it, and continue to think that everything that shows up, it could possibly be chalked up to her autism (NAE-11).”

## Discussion

In semi-structured qualitative interviews, almost all parents with and without an older child with autism expressed interest in biomarker-based testing for autism for their infant between 6-12 months of age, particularly if already concerned about their child’s development. Fewer, though still a majority of parents interviewed, were interested in predicting a child’s future support needs, and most felt that a false negative result would be more stressful than a false positive result. While attitudes across parents in both the AE and NAE groups were largely similar, the area of greatest divergence was that fewer AE parents were interested in learning about a child’s predicted level of support needs compared to NAE parents. Many of these AE parents expressed concern that a more specific prediction might limit expectations for their child in a way that could undermine intervention progress.

Parents in both groups identified the primary motivator of their interest as enabling access to early intervention, which when started prior to the age of 3 have been shown to have long-term benefits for children [20]. Importantly, however, the evidence base for autism-specific interventions started in the first year of life is nascent [4–6] and no autism-specific interventions for this age group are yet clinically available. Some parents (particularly those with autism experience) expressed awareness of this absence of services, while others conveyed the assumption that infant services are/would be available. Emerging predictive technologies are poised to accelerate the development of interventions for infants at increased likelihood of developing autism [2, 3, 9]. In the interim, however, parents who receive predictive test results could face a wait of months to years before being able to access autism-specific services, launching a “therapeutic odyssey” that requires considerable parent advocacy to leverage a predictive diagnosis into appropriate services for their child [21].

In contrast to uncertain clinical utility, parents reported clear personal utility in learning test results, such as having time to process the information emotionally, educate themselves about how to best support their child, or in some cases make large-scale changes such as renovating a home to accommodate their child’s needs or moving to an area with more access to services. As these examples illustrate, personal utility could be constrained by the resources of a family, as families with less time or fewer material resources may be less able to productively leverage the additional time afforded by a predictive test [22].

These results raise two additional considerations that should be prioritized prior to broad implementation of predictive testing for autism: First, it is important to consider how and when predictive test results should be delivered to parents. The first 12 months of life can be a challenging time for parental mental health; for example, an estimated 30-50% of mothers with postpartum depression remain affected through the first postnatal year [23]. Given the sensitivity of this period, involving appropriate professionals in delivering test results is essential. Genetic counselors are trained to deliver predictive genetic test results to families; there is not a synonymous professional group with expertise for delivering non-genetic predictive results from biomarker modalities like MRI, EEG, or eye-tracking. Empirical work is needed to establish best-practice communication tools for helping parents decide whether or not to pursue biomarker-based testing, and for supporting families after they receive predictive results [9]. Support is particularly warranted for families with low medical literacy who may be more vulnerable to non-evidence-based treatment claims and inaccurate information about autism found online [24].

Second, emerging predictive technologies raise urgent questions about how to prepare service systems for a potential influx of younger children in need of intervention services prior to an autism diagnosis. In the US, IDEA Part C federally-supported early intervention serves children age 0-3; yet in most states, children without a diagnosis, who are not yet showing evidence of delay, would not be eligible to receive Part C services [25, 26]. It is also unknown whether private and public insurance would cover predictive testing or qualify a child for autism-specific services coverage based on a predictive test. Current sociodemographic disparities in long-term outcomes for autistic children [27–30] will be exacerbated if pre-symptomatic testing and intervention is only accessible to families who can pay out-of-pocket. As recent examples illustrate [31, 32], research efforts should include cost-benefit analyses that can be used to motivate payor coverage for promising predictive tests and infant interventions as they move into clinical translation.

## Limitations

These findings likely do not capture the full range of parent attitudes towards predictive testing. Importantly, the concept of generalizability is differently considered for quantitative vs. qualitative studies [15]. We selected a sample of participants to interview not because they were intended to be representative of the broader population, but because they had specific life experiences relevant to our research questions. The insights generated from the interviews are hypothesis-generating, and suggest additional lines of inquiry, but are not definitive and we do not make claims that the attitudes of our small sample represent those of all US parents. Instead, these data are best understood in light of the particular characteristics of the sample.

AE parents were recruited from a prospective neuroimaging study, which may mean they are more interested in a predictive test (particularly one based on MRI) than other parents of autistic children. We considered the fact that this group had already completed an MRI study visit as a strength of this sample, as any parental uncertainty/anxiety about the MRI scan itself (conducted in the evening while the infant was in natural sleep) would have been resolved, leaving the interview to focus on anticipated implications of hypothetical test results, rather than the test procedure itself. That said, in future work considering clinical translation of predictive tools, the specific characteristics of a given test (hospital- or primary care-based, required time commitment, perceived safety or discomfort risk) are certainly expected to impact parental interest in testing, and warrant direct investigation.

NAE parents were recruited from a single pediatrics practice in Seattle and thus are not demographically representative of other regions in the US. There are many ways in which this group differs from the AE group aside from their lack of autism parenting experience, and thus we cannot draw generalizable conclusions about differences in all AE/NAE parent attitudes from this qualitative study. All parents completed the interview in English, and thus non-English speaking families are not represented in this sample. Finally, parents were asked to consider a hypothetical medical decision, and thus their reported attitudes might not accurately capture real-world behavior, as has been demonstrated in other applied contexts [33].

## Conclusion

Qualitative interviews with parents with and without prior autism experience revealed considerable interest in predictive testing for autism in the first year of life, largely motivated by a desire to engage in earlier intervention. Parents also shared concerns about the anticipated impacts of accurate (or inaccurate) predictive results on their child and family. These data suggest there is a broad audience for emerging technologies to predict later autism from information obtained during infancy, and pose important questions to address prior to clinical implementation related to communication of results, post-results support, and equitable provision of very early intervention.

### Supplementary Information

Supplementary Material 1. 

## Data Availability

Semi-structured interview guides are available in Supplementary materials. The full codebook used for analysis is available from the corresponding author on reasonable request. Transcript and audio recordings are not publicly available to preserve participant privacy. De-identified copies of transcripts are available from the corresponding author on reasonable request.

## Abbreviations

AE: Parents with prior autism experience (i.e., parents of an older child with autism)
NAE: Parents with no prior autism parenting experience
