# Supplementary material for: Parent attitudes towards predictive testing for autism in the first year of life
Source: J Neurodev Disord. 2024 Aug 17;16:47. doi: 10.1186/s11689-024-09561-w (PMC11330042; doi:10.1186/s11689-024-09561-w)
Supplement: Supplementary file 1 — Supplementary Material 1. [file 11689_2024_9561_MOESM1_ESM.docx]

**AE PARENT INTERVIEW GUIDE**

**Introduction**

We are interested in learning how parents in IBIS think about new methods in brain-imaging (like EEG and MRI) that are being developed to predict autism diagnoses very early, before the symptoms start. It is possible that predictive technology may be available in the next few years. We want to know what parents like you think about this possibility. There are many possible perspectives on this topic, and we are interested in the whole range of thoughts and opinions. Every opinion is valuable, and there are no right or wrong answers.

We will use the information that we learn from you today to inform future research within IBIS. We will also share what we learn with other researchers working in this field as we believe input from parents about the use of predictive technologies is critical. We want our research to be as helpful as possible to families, and we are grateful for the opportunity to talk today and learn about your experience and perspective.

Do you have any questions about the purpose of this call?

Is it ok with you if I begin recording our call at this point?

(Wait for affirmative or stop interview).

**Warm-up**

To start, can you tell me why you decided to enroll your child in the IBIS study?

It is common for people participating in research to have a variety of hopes and expectations about what will come their participation.

Let’s start with what you optimistically hope will happen….

*For you/your family?*

*For scientific knowledge?*

Now please tell me what you realistically expect will happen (if that is different)…

*For you/your family?*

*For scientific knowledge?*

What has the experience of being in the IBIS study been like so far?

[If received feedback from developmental assessment]

*What did you learn?*

*What was that like for you?*

[If not already mentioned] What do you know about [CHILD’S NAME]’s likelihood of developing autism?

**Predictive testing for ASD**

Imagine that it were possible to use brain imaging at 6-12 months to predict which infants are likely to develop autism. [CHILD’S NAME] is currently [AGE] months old, is that right? I’d like you to imagine that right now we could tell you whether he/she is likely to develop autism. Would you want to know this information?

*Why?*

*Why not?*

*Would your spouse/other family members agree?*

*What might be some advantages of learning this information?*

*What might be some disadvantages?*

*What might be some reasons that other families would make a different choice?*

*One limit of the test is that it might not be able to tell you where on the spectrum your child is likely to fall, or what level of support needs they will have. Would it be different if the test could tell you about the future support needs?*

Imagine that you learned that [CHILD’S NAME] was not likely to develop ASD. What do you think your reaction would be?

*How would it affect you? Your family?*

*How would it affect your relationship with your child?*

*Is there anything you might do or change as a result?*

Imagine instead that you learned that [CHILD’S NAME] was likely to develop ASD. What do you think your reaction would be?

*How would if affect you? Your family?*

*How would it affect your relationship with your child?*

*Is there anything you might do or change as a result?*

[if not already mentioned] *Would you seek out intervention?*

Most tests that predict diagnoses are not 100% accurate. This means that a predictive result could be wrong—for example, you could be told that your child is going to develop autism, when in fact he/she would develop typically.

*What would that be like for you?*

*How do you think you and your family would handle that situation?*

The opposite could also occur—you could be told that your child is not going to get autism, when in fact he/she will. How do you think you would manage that situation?

*What would that be like for you?*

*How do you think you and your family would handle that situation?*

**Closing**

We’ve talked about some important things today, and I really appreciate you sharing your thoughts with me. Is there anything else you’d like to share? Is there anything I haven’t asked about that you’d like me to know?

Thank you so much for speaking with me.

**NAE PARENT INTERVIEW GUIDE**

**Introduction**

We are interested in learning how parents think about new methods that are being developed for assessing infant development in the first year of life. Examples of such methods are neuroimaging techniques like MRI and EEG. There are many possible perspectives on this topic, and we are interested in the whole range of thoughts and opinions. Every opinion is valuable, and there are no right or wrong answers.

We will use the information that we learn from you today to inform future research. We will also share what we learn with other researchers working in this field as we believe input from parents like you is critical. Any information we share from you and other families will be fully confidential. We want our research to be as helpful as possible to families, and we are grateful for the opportunity to talk today and learn about your experience and perspective.

Do you have any questions about the purpose of this call?

Is it ok with you if I begin recording our call at this point?

[Wait for affirmative or stop interview]

**Warm-up**

To start, I’d love to hear a little a bit about your family. You can just use general descriptors to tell me (like son, partner, sister) rather than names. Who do you live with?

Have you had experience with any developmental checklists? Maybe in your pediatricians office?

*What was that like? What did you learn?*

One of the goals of checklists that are often used in the doctor’s office (though sometimes not until older like 18 months) are for detecting symptoms of specific neurodevelopmental disorders like autism. Do you know anyone who has autism?

What do you know about autism? (it’s ok if not much)

*What does it look like?*

*At what age does it start?*

*How common is it?*

**Predictive testing for ASD**

Researchers are developing tools brain imaging at 6-12 months to predict which infants are likely to develop autism. I’d like you to imagine that right now we could tell you whether [CHILD NAME] is likely to develop autism. Would you want to know this information?

*Why?*

*Why not?*

*Would your spouse/other family members agree?*

*What might be some advantages of learning this information?*

*What might be some disadvantages?*

*What might be some reasons that other families would make a different choice?*

*One limit of the test is that it might not be able to tell you where on the spectrum your child is likely to fall, or what level of support needs they will have. Would it be different if the test could tell you about the future support needs?*

When you think about this decision, does it feel similar or different to other medical decisions you’ve made for your child, or for your own health?

Imagine that you learned that [CHILD NAME] was not likely to develop ASD. What do you think your reaction would be?

*How would it affect you? Your family?*

*How would it affect your relationship with your child?*

*Is there anything you might do or change as a result?*

Imagine instead that you learned that [CHILD NAME] was likely to develop ASD. What do you think your reaction would be?

*How would if affect you? Your family?*

*How would it affect your relationship with your child?*

*Is there anything you might do or change as a result?*

[if not already mentioned] *Would you seek out extra support or intervention? If so, what?*

Most tests that predict diagnoses are not 100% accurate. This means that a predictive result could be wrong—for example, you could be told that [CHILD NAME] is going to develop autism, when in fact he/she would develop typically.

*What would that be like for you?*

*How do you think you and your family would handle that situation?*

The opposite could also occur—you could be told that [CHILD NAME] is not going to get autism, when in fact he/she will. How do you think you would manage that situation?

*What would that be like for you?*

*How do you think you and your family would handle that situation?*

**Closing**

We’ve talked about some important things today, and I really appreciate you sharing your thoughts with me. Is there anything else you’d like to share? Is there anything I haven’t asked about that you’d like me to know?

Thank you so much for speaking with me.
